# *Active* and *reactive* behaviour in human mobility: the influence of attraction points on pedestrians

**DOI:** 10.1098/rsos.160177

**Published:** 2016-07-13

**Authors:** M. Gutiérrez-Roig, O. Sagarra, A. Oltra, J. R. B. Palmer, F. Bartumeus, A. Díaz-Guilera, J. Perelló

**Affiliations:** 1 Departament de Física Fonamental, Universitat de Barcelona. Martí i Franqués 1, 08028 Barcelona, Spain; 2 Universitat de Barcelona Institute of Complex Systems UBICS, 08028 Barcelona, Spain; 3 Centre d’Estudis Avançats de Blanes (CEAB-CSIC), Accés a la Cala Sant Francesc, 17300 Blanes, Spain; 4 Centre de Recerca Ecològica i Aplicacions Forestals (CREAF), Campus de Bellaterra (UAB) Edifici C, 08193 Cerdanyola del Vallès, Spain; 5 ICREA, Passeig Lluis Companys 23, 08010 Barcelona, Spain

**Keywords:** computational social science, behavioural experiments, human mobility, random walk

## Abstract

Human mobility is becoming an accessible field of study, thanks to the progress and availability of tracking technologies as a common feature of smart phones. We describe an example of a scalable experiment exploiting these circumstances at a public, outdoor fair in Barcelona (Spain). Participants were tracked while wandering through an open space with activity stands attracting their attention. We develop a general modelling framework based on Langevin dynamics, which allows us to test the influence of two distinct types of ingredients on mobility: *reactive* or context-dependent factors, modelled by means of a force field generated by attraction points in a given spatial configuration and *active* or inherent factors, modelled from intrinsic movement patterns of the subjects. The additive and constructive framework model accounts for some observed features. Starting with the simplest model (purely random walkers) as a reference, we progressively introduce different ingredients such as persistence, memory and perceptual landscape, aiming to untangle *active* and *reactive* contributions and quantify their respective relevance. The proposed approach may help in anticipating the spatial distribution of citizens in alternative scenarios and in improving the design of public events based on a facts-based approach.

## 1. Introduction

The development of information and communications technologies (ICT) is changing the way we interact with the world, and by extension the way we perform science. We have entered the big data era [1] which, in principle, puts in the hands of researchers enormous possibilities to monitor, study and understand human activities. Among such technologies, the development of readily available, cheap and reliable geolocalized devices has fostered the field of studies in human mobility. Computer vision analysis [2], Bluetooth devices [3,4], radio-frequency identification (RFID) signal intensity [5,6], mobile phone calls [7–9] and geolocalized social network feeds [10] represent just a handful of the methods and data sources that have now been successfully exploited for this purpose.

Despite the recent technological advances, two important challenges remain for the scientific community: accessibility and data biases. On the one hand, data openly available for human mobility research are scarce and somewhat restricted. ICT companies are reluctant to freely share their data with the scientific community, and when they do so, it is often with strict constraints that pose challenges for the reproducibility of experiments. On the other hand, human mobility data gathered directly from traditional research volunteers are often limited by privacy considerations and scalability. In this paper, we propose that the emerging citizen science research model [11–13] offers us an alternative strategy that can solve the problems of data quality, control, property rights, accessibility and privacy, offering reproducible results. We use this strategy to investigate human mobility at an outdoor science fair in Barcelona (Spain). By gathering location information in a public space and making volunteers active participants in the scientific process, we are able to easily construct a high-resolution and open-access dataset.

In the context of human mobility, cell phone GPS receivers offer us a good technological option for gathering location information [14], one that also bridges the field of human movement in open spaces with that of behavioural ecology [7,15,16], with its rich and consistent literature on home-ranging and foraging. As an alternative approach to the existing literature [7,15,17–20], we thus here present the results of an experiment on a microscopic scale [21] where participants were invited to wander around an open space containing the fair’s activity stands while tracking themselves using a mobile phone application. Individuals could freely move around and access any of the stands. The space in which they moved was not isotropic—it included obstacles, paths, trails and forbidden walking areas—but the stands were set up to clearly attract most of the attention and drive movement dynamics in this ludic, open-air event.

A key aspect to understanding organisms’ movement patterns, including those of humans, is determining how *active* and *reactive* behavioural components are exploited depending on scales and information availability [22–24]. Depending on the state of the organism (e.g. level of hunger, stress) and the amount and quality of the available information, movement is affected by sensory or memory information (*reactive* motion) or else by a more explorative inherent component (*active* motion). Importantly, exploratory movement may also be guided by sensors and cognitive processing (past experiences), but the motor connections between cause and effect should be considered less explicit and time-delayed. *Reactive* movement is then more promptly associated with movement driven by external triggers, whereas *active* motion is more likely context-independent and internally driven [22–24]. One may also connect the binary *reactive*/*active* distinction to the classic debate in movement ecology on whether external or internal factors govern movement [25]. In any case, the distinction between *reactive* and *active* motion should be taken cautiously and in relative terms, with *active* motion viewed as involving more free movement actions than *reactive* motion, but not being the exclusive domain of such actions.

Here, we model and characterize human movement at the Barcelona science fair in terms of *active* and *reactive* components. We propose a framework model based on Langevin dynamics [26–28] to characterize participants’ motion to account for some of the observed features. Starting with the simplest model, pure random walkers (RW), as a reference, we progressively introduce different ingredients such as persistence, memory and perceptual landscape [29], aiming to untangle *active* and *reactive* contributions and quantify their respective relevance. We then compare the limitations and strengths of the proposed models, and discuss the delicate balance between complexity and accuracy when modelling human movement. The framework we propose is flexible enough and sufficiently easy to handle to be used as a tool to better understand mobility in other contexts.

## 2. Results

### 2.1. The experiment, the data and some basic definitions

**Figure 1.**
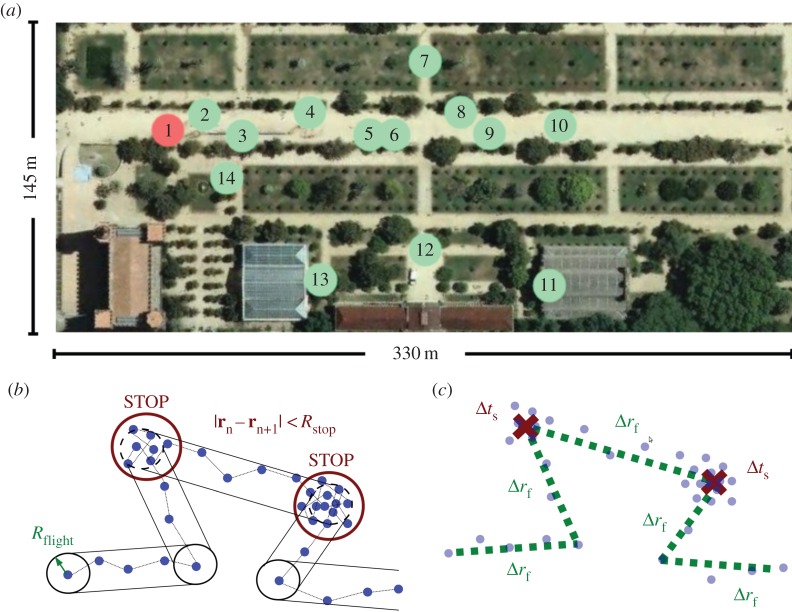
Map of the fair indicating location of stands and stops and flights discrimination procedure. (*a*) Stand number one, coloured in red, shows the location of Bee-Path welcoming location, where participants were recruited. This image is produced from an orthophoto of ‘Institut Cartogràfic i Geològic de Catalunya’ under a Creative Commons license CC-BY. (*b,c*) Each blue spot represents a GPS position recorded with a given time-stamp. Stop-and-run algorithm described in Methods is applied detecting the red circles of radius *R*_stop_ as stopping points whose duration is Δ*t*_s_. Then, rectangular grid criteria whose width is calibrated with parameter *R*_flight_ (*b*) detects in this case five different flights with Euclidean lengths Δ*r*_f_ (*c*).

We carried out the experiment, called Bee-Path (BP), at Barcelona’s annual science festival, a major event held in an public park and promoted by the city council. During two consecutive days, around 10 000 visitors attended to the fair, some of them were tracked as they wandered around the park to visit fair stands that offered a variety of activities. While the participants had access to some information about the activities held in the stands (and possibly some prior knowledge about the environment), they were recruited upon entering the space, as shown in figure 1, and hence are assumed to have had no previous direct experience in exploring the fair.

We succeeded in engaging 101 participants recruited at different moments of the event. After removing the outliers from the data (see the electronic supplementary material), we ended up with 4994 GPS locations, corresponding to 27 participants and roughly 27 h of experiment. The time-stamped GPS locations for each subject were converted into individual tracks decomposed in a collection of *flights* and *stops* by using a two-step procedure (see Methods and figure 1): first, locations were flagged as either *moving* or *stopped*. Second, successive locations flagged as *stopped* were used to determine stop duration, and the successive locations flagged as *moving* were grouped into flights using the so-called rectangular grid criteria [30] (see figure 1 for further details). Each stop has a time duration Δ*t*_s_ and a location **x**_s_ defined as the average position of its stopped points. Conversely, each flight has a time duration Δ*t*_f_ and a length Δ*r*_f_ defined as Euclidean distance between starting and ending points. These two magnitudes also allow us to obtain an average velocity for each flight *v*=Δ*r*_f_/Δ*t*_f_. Each participant has a track defined by these flights and stops, and each track has an associated mean velocity, calculated as the average of its flight velocities. There are a total of 350 flights and 300 stops.

Although the participants vary in demographic characteristics, we find no particular profile in terms of age or gender and we assume population homogeneity, pooling all data to improve statistical discernment between *stopping* and *moving* states, better detect main orientation features, and minimize the effect of location errors caused by GPS noise (see discussion in electronic supplementary material). Also crucial is the assumption that the stop-and-run tracks obtained adequately characterize key aspects of the actual movement, which may be intrinsically assumed to be a continuous process in space and time. The most we can do is to consider and evaluate potential biases in the conversion from discrete GPS locations to stop-and-run walks by exploring how parameter choices in the movement processing algorithm affect results (see electronic supplementary material, where also the biases on an isotropic RW are analytically evaluated).

Considering these preliminaries, we analyse movement features from a twofold perspective. We first assess participants’ overall space use by computing the population-level utilization distribution. Second, we take the stop-and-run walks approach to compute movement metrics that help us to understand key features of movement as well as to evaluate how the empirical data fits into different classes of proposed models.

### 2.2. Movement patterns

**Figure 2.**
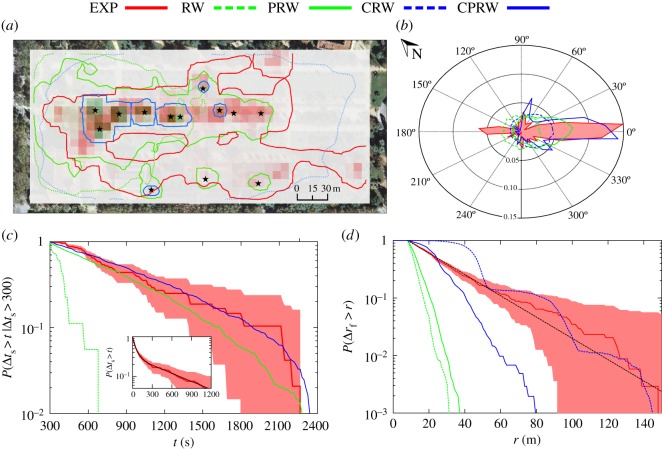
Empirical results (EXP, red) compared with dynamics of each Bee-Path framework model: RW (green dashed), correlated random walk (CRW, blue, dashed), potential-driven random walk (PRW, green, solid), and correlated potential-driven random walk (CPRW, blue, solid). (*a*) The dBBMM utilization densities, shown as coloured raster cells (2.5 standard deviation colour stretch, with darker colours indicating higher use intensities) and as 95% isopleths, overlaid on ortophotomap of Ciutadella Park from ‘Institut Cartogràfic i Geològic de Catalunya’. Attraction wells (stands) indicated by stars. (*b*) Flight orientation polar plot where radial component measures the probability at the corresponding angle. RW, CRW, PRW, CPRW jointly with the results from the empirical flights. (*c*) The stop duration complementary distribution function (CDF) for long stops (Δ*t*_s_>300 s) is calculated for each dynamics with a bin size of Δ*t*_s_=15 s. Shaded area around the real curve represents the cumulative standard deviation calculated as $\sqrt{\sum_{0}^{i}p_{j}(1-p_{j})/N}$, where *p*_*j*_ is the value of bin *j* and *N* is the number of stops. The inset shows the complete CDF stop duration distribution for the real time and the analytical function (black solid line) with *ω*=0.308±0.008, *τ*_1_=617±17 s and *τ*_2_=37.5±1.5 s ($\chi_{\mathrm{red}}^{2}=6.57\times 10^{-5}$). (*d*) Flight length CDF is calculated and dashed black line corresponds to heuristic fit with λ=27±2 m ($\chi_{\mathrm{red}}^{2}=0.008$). The CRW shows a bumpy behaviour, because of the combination of time discretization process (every 15 s) and directional correlation of the model.

We compute the population-level utilization distribution in two-dimensional space using a dynamic Brownian bridge movement model (dBBMM) [31–33], which assumes observed locations are bridged by Brownian motion, with the diffusion coefficient adjusted for each bridge based on observed variance in the trajectory. We apply this model to all tracks to calculate, for each cell in discretized observation space, the relative frequency of participants’ presence during the experiment (see Methods for further details). Figure 2*a* shows that participants were mainly concentrated around the stands, albeit with this concentration seeming to decrease as we move further away from the starting point of the experiment. Therefore, the specific configuration of the stands, placed along the *x*-axis, has a strong effect on the mobility of the participants. The space shows the non-isotropic character of urban areas and spaces, which, in many cases, is purposeful (stand distribution and accessibility were deeply discussed by the fair’s organizing team). The polar plots in figure 2*b* further confirm this finding: the flight orientation is biased in the direction of the promenade—the *x*-axis—where the concentration of stands is high, whereas the asymmetry between the positive, 0, and negative direction, 180, in the *x*-axis reveals the general tendency of the people to form a flow from the entrance of the park, at the left of the map, to the last stand of the fair, at the right. We also observe a backwards flow generated by those participants that after having visited the fair, exited through the initial entrance or by those that returned to our stand to see their tracks.

The empirical stop duration statistics can be observed in figure 2*c*. The observed stop duration complementary distribution function (CDF) can thus be well fitted by a weighted double exponential law with two different characteristic decaying times

2.1 $$\begin{array}{r} P(\Delta t_{s}>t)=\omega \exp\left(-\frac{t}{\tau_{1}}\right)+(1-\omega )\exp\left(-\frac{t}{\tau_{2}}\right). \end{array}$$

**Figure 3.**
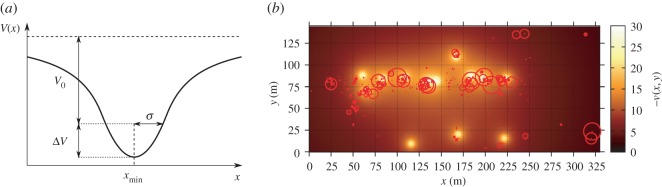
Proposed gravitational wells representing attractiveness of fair stands. (*a*) Section across *x*-axis direction of one potential well. (*b*) Heat map of fair’s potential landscape, with potential wells placed at the fourteen stands locations. All participants’ stops indicated by red circles with radius proportional to stop duration. Note that longest stops closely coincide with stand locations.

The shortest characteristic decay time is around 30 s and corresponds to reorientation processes. As shown in figure 3, the shortest stops (smaller circumferences) appear to be distributed at the central part over the fair space but not only concentrated around the stands, indicating that this time scale may be related to an orientational process not directly driven by the fair stands (*reactive* behaviour), but also linked instead to other factors. In contrast, figure 3 also shows that the longest stops are much closer to fair stands (spots in yellow) where activities are happening. The characteristic time scale of these stops is around 10 min (largest characteristic scale in the CDF of equation (2.1)). We note that such characteristic stop duration is much smaller than the time span of the activities held in the stands, suggesting limited attention capacity [34] by the fair participants. The fair included science outreach activities, such as talks and hands-on workshops, with durations generally between 30 min and 1 h or in some cases even longer. Some stands hosted activities with no specific duration, lasting an entire morning or afternoon, or even the whole day. Based on the divergence between the time length of the stops at the stands and the length of the activities prepared for the fair, we have advised the organizers to aim for shorter, fresher and more dynamic activities to better capture audience attention in future editions of the fair.

Figure 2 also shows that the flight length CDF displays a single exponential decay

2.2 $$\begin{array}{r} P(\Delta r_{f}>r)=\exp\left[-\frac{\left(r-R_{\mathrm{stop}}\right)}{\lambda }\right]\;\text{if}\;r>R_{\mathrm{stop}}. \end{array}$$

We note that the distribution has a natural lower bound when *r*=*R*_stop_ forced by the rectangular grid criteria for the flights algorithm described in figure 1*b,c*. Owing to the relatively small park dimensions and moderate densities, one could neither expect very large displacements nor identify heavy-tailed flight behaviour [7]. Results show, however, a relatively slow decay compared with the overall scale of the fair space (330×145 m), with a characteristic length of λ=27 m. This scale is large enough to consider the presence of such long flights as unexpected, given the moderate densities and the small and bounded environment in which fair activities were taking place. Our experimental data show that some participants skipped stands, sometimes making larger flights more probable, and exhibiting the type of short-range directional persistence commonly found in animal movement [35]. An extended discussion is provided in the electronic supplementary material.

A final feature worth studying is the distribution of flight velocities. This is highly peaked around 0.6±0.3 m s^−1^ which is below standard human velocity speed. The small average flight velocity detected is probably the result of a moderate density of attendants to the fair in combination with the multiple activities available to visitors. Moreover, such a reduced variance in flight velocity also suggests low individual variability and the presence of a homogeneously behaved population. We computed velocity distributions discarding the stops, only using the moves or flight segments.

### 2.3. The Bee-Path attractivity model framework

Pollinators, such as bees, move across the landscape looking for resources (within flowers) by following a stop-and-run process [22,35]. The resulting stop-and-run paths emerge as a combination of *reactive* (extrinsic) and *active* (intrinsic) individual behaviour. Similarly, the human movement observed at the fair can be modelled as a stop-and-run process driven by two main components: external inputs and intrinsic motion. Individuals largely wandered through the fair according to their instincts or some vague expectations (context-independent decisions) but, from time to time, they could *feel the attraction* of specific stands hosting specific activities. We develop here minimal models of increasing complexity (BP model framework) that may help us to distinguish external from internal drivers of motion, and define the key features of the movement observed within the fair.

To describe the position **r**(*t*) of an individual at time *t*, we use a two-dimensional stochastic model based on Langevin dynamics [26], which describe the trajectory of a particle moving randomly owing to a thermal bath (*internal energy*) and subject to a field of forces (*external energy*). We assume individuals have no inertia: although humans clearly have memory of past events, our assumption here is that their orientation decisions during the fair are mostly taken with respect to actual circumstances [36]. The dynamical equation for the BP model framework, discretized in intervals of Δ*t* and in the Itô sense [37] then reads

2.3 $$\begin{array}{rl} \text{v}(t) & =\frac{\text{r}(t+\Delta t)-\text{r}(t)}{\Delta t}=\frac{1}{\gamma }\nabla V(\text{r}(t))+\sqrt{\frac{2}{\gamma \beta \Delta t}}\rho \hat{\text{u}}(\theta (\kappa ,\Delta t))=\frac{1}{\gamma }({\text{F}}_{\text{R}}+{\text{F}}_{\text{A}}),\;\hat{\text{u}}(\theta )=(\cos\theta ,\sin\theta ), \end{array}$$

where the first term stands for the potential landscape capturing the field of forces (*reactive* component, **F**_**R**_), whereas the second refers to the random fluctuations on the movement (*active* component, **F**_**A**_). Drag parameter *γ* is manually set to unity following the assumption that all individuals are equally susceptible to the action of both the *reactive* and *active* forces (a change of this parameter would only redefine the time scale of the process).

The *active* component, stochasticity of equation (2.3), is represented in polar coordinates. The radial part is determined by a random variable *ρ* that controls the modulus of the velocity, distributed as $p(\rho )=\rho \exp(-\rho^{2}/2)$, and multiplied by a term governed by parameter *β* that governs the amplitude of the fluctuations. The angular part is described by the random variable,

2.4 θ(t+Δt)=θ(t)+Θ,

whose difference with the previous step follows the Von Mises distribution [36]

2.5 $$p(\Theta )=\frac{1}{2\pi I_{0}(\kappa /\Delta t)}\exp\left[\frac{\kappa }{\Delta t}\cos(\Theta )\right],$$

where *I*_0_ is the modified Bessel function of order 0 and *κ* accounts for the persistence of the movement. Note that when *κ*=0, we recover an RW, and conversely, when *κ* increases, the probability density function becomes sharper around zero introducing reinforcement in the movement orientation and thus obtaining the correlated random walk (CRW) dynamics.

In the *reactive* component, the potential landscape *V* (**r**) is generated by the joint effect of fourteen different sources of identical shape, which are located in the same positions as the stands in the experimental set-up (figure 1). Each single stand is represented as a gravitational-like attractive potential well generated by a non-punctual spherical attractive mass [37]

$$V(\text{r}|{\text{r}}_{0})=\left\{\begin{array}{ll} -V_{0}\frac{\sigma }{|\text{r}-{\text{r}}_{\text{0}}|} & \text{if }|\text{r}-{\text{r}}_{\text{0}}|\geq \sigma \\ -\frac{3}{2}V_{0}+\frac{1}{2}V_{0}\frac{|\text{r}-{\text{r}}_{\text{0}}{|}^{2}}{\sigma^{2}} & \text{if }|\text{r}-{\text{r}}_{\text{0}}|<\sigma . \end{array}\right.$$

The two constants of the potential are fixed in terms of the radius of the non-punctual spherical region *σ* (related to the size of the stands) and in terms of the intensity of the potential outside this circular region *V*_0_ (related to the attraction force). Intensity solely depends on the Euclidean distance |**r**−**r**_**0**_|. We therefore construct a set of attractive wells located at each of the stands of the fair as shown in figure 3. In our case, for the sake of simplicity, both *σ* and *V*_0_ are taken as equal for all stands, with the intensity of the stands’ *V*_0_ adjusted from real data, and *σ*=4 m chosen according to the actual physical area of the original stands. Although our model does not consider the possible differences of *attractivity power* among stands, one could easily implement different *V*_0_ and *σ* pairs for each stand, inferred from the observed relative importance in the data.

We consider an individual to be trapped by an attractor when she approaches a given stand for the first time and comes within a distance shorter than *σ*; the other potentials then deactivate, representing that her attention is fully focused on the activity of that stand. Under this scenario, we observe that the *stopping* locations of the motion dynamics can be described by the classic Kramers problem [38] extended to two dimensions, which evaluates the statistics of the trapping times. The classic exponential decay distribution of the Kramers problem is consistent with empirical stop duration distribution (longest stops) as shown in figure 2. The ratio of parameters *V*_0_ and *β* determines the decaying ratio in the distribution of stops (see electronic supplementary material for a complete exploration of the parameters space). To incorporate memory effects in our modelling approach, we consider that after an individual has visited one of the attractive wells (a stand) and leaves it (being further than distance *σ*), the subject ceases to feel its attraction and henceforth, the attractive well is deactivated for this walker.

Therefore, the motion in our experiment is described by equation (2.3) with the potential *V* (**r**(*t*)) taking the explicit forms in equation (2.6) depending on the position of the subjects. They will be most of the time in a *moving* state described by the first expression corresponding to |**r**−**r**_**0**_|≥*σ*. Occasionally, subjects are *trapped* inside a potential well described by the second expression. Therefore, dynamics are analogous to the Kramers problem [38] and our processing algorithm labels them in a *stopping* state. We thus see how this description bridges the *stop-and-run process* from behavioural ecology to the continuous in time BP model framework.

Our approach implicitly assumes no interaction among individuals, so disregards crowd effects as a first level of description. These effects, however, could be added in the form of additive pairwise forces among users [2,26,27]. In these models, the random forces [26] consider first a term describing the acceleration towards the desired velocity of motion (that might be somehow comparable to our *active* component); second, terms reflecting that a pedestrian keeps a certain distance from other pedestrians and borders (we discard this contribution in our analysis) and third, a term modelling attractive effects (that might be somehow comparable to our *reactive* component). Moreover, our model has also dismissed the effect of obstacles or any other non-human objects that certainly have a fundamental role in crowd dynamics [2,39].

### 2.4. Shaping the *active* and the *reactive* passive components

The BP model framework proposed in equation (2.3) is highly flexible and makes it possible to clearly identify the basic ingredients of movement in the fair, recovering some of the empirical features of the experimental data. The model used contains a maximum of four free parameters (*κ*, *β*, *V*_0_ and *σ*) although *σ* is defined by the geometry of the attraction wells. We thus fix *σ*=4 m.

We can disentangle different aspects by playing with free parameters of the BP model framework and then simulate the subsequent four possible types of dynamics. First, we can consider a pure RW without attraction wells, so that individuals have only an *active* component (*V*_0_=0). Second, we can test another purely *active* scenario (*V*_0_=0), where the only new ingredient is a persistence (*κ*) on keeping the direction of each previous step with a non-isotropic noise. This case is known as a correlated random walk (CRW) in the literature [36]. These two scenarios are considered as null models in order to capture the effect of introducing a potential landscape. Then, in a third scenario, we can observe a different situation where the individuals *feel* the attraction wells, but the underlying movement is a RW such that *active* and *reactive* components are present (*κ*=0 while *V*_0_ and *β* are free parameters). We call this case potential-driven random walk (PRW). Finally, we can combine both persistence and potential landscape (*κ*, *V*_0_, *β* are free parameters) in what we call correlated potential-driven random walk (CPRW). We have selected the values of the parameters for the PRW (*V*_0_ and *β*) and CRW (*V*_0_, *β* and *κ*) models by optimizing their performance when looking at the empirical stop statistics as carefully discussed in the electronic supplementary material. The RW and CRW models take the same values as those from the equivalent models with potential attractive wells (PRW and CPRW).

The presented framework aims to study and identify the minimum ingredients necessary to capture the essential traits of human mobility at the scales studied. By means of hypothesis testing using the different proposed dynamics, we are able to assess the effect each additional parameter has on the observed movement patterns. The resulting statistics for each scenario are shown in figure 2. Both the RW and CRW cases are obviously taken as null models. Lacking spatial coherence, they are unable to reproduce the spatial usage and orientation of the subjects or their stopping statistics. It is noteworthy, however, that the RW dynamics generate a flight velocity distribution (that of a Maxwell–Boltzmann distribution, see the electronic supplementary material) close to the one observed in empirical data, indicating that a random noise term is a necessary addition to the model. As expected, the addition of correlation in the consecutive updates of the movement direction (CRW) leads to long flights (with an almost constant velocity), yet very large values of correlation are needed in order to partially reproduce the tail of the flight distribution (and the results obtained are not smooth due to the fact that all flights generated are excessively rectilinear, and this generates bias on the movement processing algorithm, see the electronic supplementary material).

Adding the presence of potential wells significantly increases the accuracy of the model, validating the hypothesis of perceptual landscapes [29] and stressing the need of including *reactive* components. Both the spatial distribution and temporal statistics of stops are recovered (with two distinctive time scales), and the flight’s velocities are again reproduced. The flight orientation is also distinctively shifted towards the direction of the fair promenade (although the significant tendency of users to return to the origin in the opposite sense is not captured by the model, as it does not consider memory effects). The presence of long flights and the distribution of flight orientation, however, cannot be solely explained by the presence of attraction wells, because the absence of correlation in the noise term severely limits the probability of long displacements, which as discussed earlier is a distinctive feature of the observed data. Long flights cannot be accounted for by the dynamics presented, and are probably a result of a combination of interaction effects between individuals and the crowd, and directional bias owing to the existence of a preferred direction imposed by the fair’s main promenade. Confirmation of this fact is given by the CPRW dynamics, which include attraction and correlation effects, yet are still unable to capture the abnormally long (but not heavy-tailed) detected flights. Moderate improvements are detected on flight orientation and length distribution, yet the short scale of the stop statistics is lost. Even adding an extra ingredient accounting for selection of destinations by each individual—a probability for each potential well not to be considered by the individual—worsens the predictive power of the model (see the electronic supplementary material).

Our approach links the two different contributions (*active* and *reactive* component) to the dynamics in a straightforward way. Furthermore, our approach through equation (2.3) makes it possible to quantify the balance between the competing components as expressed by the relative contribution to power developed by each of the acting forces **F**_R_ and **F**_A_, which can be evaluated through the work generated by each of the two (*reactive* and *active*) forces (see the electronic supplementary material). The PRW displays a ratio 70/30% between the components, whereas the CPRW only has a 3% *reactive* contribution, which nevertheless is capitally important to reproduce spatial features of the walk.

## 3. Discussion

This work explores the subject of human mobility in mid-range and non-crowded but mildly dense environments, from both empirical and theoretical perspectives by analysing and modelling the results of an experiment with pedestrians. The main contribution of this paper consists of elucidating the minimal ingredients necessary to reproduce human mobility features in the context of a fair, where external and internal motion drivers are at play.

Our modelling approach proposes an additive, constructive and simple framework where pedestrians *feel* the attraction of a collection of points of interest located in the fair. Starting with the most simple model (purely RWs), we progressively introduce persistence and perceptual landscape (with attractive potential wells) aiming to untangle *active* (inherent factors) and *reactive* (external) contributions by only adding up to three parameters in the model (table 1). The combination of persistence and perceptual landscape under a Langevin dynamics framework [26] succeeds in explaining most important observed aspects in this context: the non-isotropic character of the human motion, the exponential decay of stop duration statistics owing to the attraction of activities in stands, the exponential decay in flight lengths statistics during movement phases (finding a highly peaked velocity distribution). The framework is also able to quantify the relative importance of *reactive* and *active* components, showing that even a relatively small and subtle *reactive* contribution can describe the basic traits of human motion in these contexts.

**Table 1.** Overview of characteristics and agreement with empirical data for random walk (RW), potential-driven random walk (PRW), correlated random walk (CRW) and correlated potential-driven random walk (CPRW) models. ‘Dynamics’ columns indicate whether dynamics are driven by a potential and whether they incorporate persistence. ‘Observables’ columns compare each model’s dynamics with empirical utilization density, flight orientation and stop and flight complementary distribution functions (cf. figure 2), using (−) to indicate disagreement, (+) moderate agreement and (++) good agreement. ‘Components’ columns measure the importance of *reactive* and *active* components in each case (see the electronic supplementary material for further details). Note that PRW shows a slight tendency to flight orientation, but less intense than CPRW, whereas CPRW is able to reproduce flight distribution when probability of skipping wells is introduced in the model as discussed in the electronic supplementary material. As also shown in the electronic supplementary material, velocity distribution of CPRW is wider and larger (median and average) than that empirically observed while PRW better coincides with observations.

	dynamics		observables reproduced				components	
	potential	persistence	util. dens.	fl. orient.	stops	flights	*active* (%)	*reactive* (%)
RW	no	no	−	−	−	−	100	0
PRW	yes	no	+	+	++	−	32	68
CRW	no	yes	−	−	−	+	100	0
CPRW	yes	yes	+	++	+	+	97	3

Directional memory effects are apparent in the way the different stands are visited, whereas a limit to the attention capacity of the participants in the fair is observed in the large-scale regime of about 10 min present in the stop duration statistics. The influence of the environment is patent from the spatial concentration around attention poles (attraction wells) and orientation of exploring patterns observed in the data. The presented framework is flexible enough to accept further enhancements. It would be possible, for instance, to add extra ingredients to account for destination selection or interaction with the crowd [26,39]—which might better explain the longest flights or the strong homogeneity in participants’ velocities. We limit ourselves here to examining how the CPRW model might be extended to look at the destination selection effect, leaving other potential enhancements for future work.

Our modelling approach contrasts that of continuous time random walks (CTRWs) [40], because it aims to describe movement, which is an inherently time-continuous process, emerging from the interaction of several well-defined factors such as the presence of potential attractive wells [37]. It then allows us to test whether observed features from the data (such as stop duration statistics) can be recovered under the hypotheses of the model, furthermore, it allows us to explore the effects of data treatment and discretization and understand their biases. Last but not least, it further permits us to quantitatively evaluate the balance between these components by means of physical magnitudes owing to the straightforward interpretation one can extract from their computation.

Our intention when using the Langevin dynamics framework is to disentangle user-dependent factors from environmental ones. The contribution of the random force, whose parameters could be selected from a given population distribution if needed, are aimed at capturing subject-specific factors and thus are independent of space, state of movement or other environmental factors. Obviously, the psychological factors that determine the choice of each direction are very difficult (if not impossible) to model, yet, one can use a simplified view by assuming that its averaged outcome results in a stochastic force of the type captured by the Langevin dynamics framework. Needless to say, this interpretation is not analogous to the usual one for random movements of particles in fluids [26], but our approach allows us at least to establish clear hypothesis testing on the factors that determine the spatial and temporal lengths of movements, and how they are obtained (assessing also the influence of the filtering algorithm used) from a time-continuous process, rather than imposing them externally as the CTRW framework usually does. On the one hand, the Langevin framework also allows us to easily introduce crowd effects in the contribution of the (non-stochastic) attraction forces as pairwise interactions (or approximate interactions with the potential depending on the occupation of each stand) [2,39].

Despite the success of the models in reproducing some of the observed features, the bimodal nature of these types of movement (stop-and-run states) together with human cognitive factors determining stand destination selection present a severe limitation to their description by means of a time-continuous stochastic framework. Simple models such as the one presented here cannot account for complex factors that determine human mobility at an individual level. Notwithstanding this, the models presented can be used to reproduce a variety of real-world situations such as movement through parks, fairs, exhibition rooms and many other relatively small spaces with a medium–low density of individuals. The BP framework is flexible enough to easily modify the location of poles of attention (attraction wells) and then simulate the resulting pedestrian movements as people are attracted to them. This flexibility can be of great interest for redesigning spaces or anticipating pedestrian mobility in alternative spatial distributions.

Human mobility is a very complex process involving cognitive, physical and socio-economic factors, as well as a mix of spatial and temporal scales [15]. Moreover, despite the efficiency of the different technologies used for tracking purposes, different technical limitations arise in each particular scenario, making its study an interesting cross-disciplinary area where technical, experimental and theoretical challenges from very different disciplines can meet. This paper presents the results of one such cross-disciplinary collaboration, where modelling of human-related mobility in short ranges is covered. The junction of the open culture embedded in citizen science together with the endless possibilities in the advances in information technologies opens new and interesting opportunities such as the one exploited here. We hope that more experiments will follow and they will help tackle the challenges that the study of human mobility in medium ranges present.

## 4. Methods

### 4.1. Experimental setting

During the weekend of 16 and 17 June 2012, the Institut de Cultura de Barcelona (Culture Institute of the Barcelona City Council) organized the Festa de la Ciència i la Tecnologia (Science and Technology Fair). It is considered to be the most important annual science outreach event in Barcelona for general audiences. In 2012, it was organized as a set of 14 stands and buildings located in an area of more than 4 ha (330×145 m) inside the public green park called Parc de La Ciutadella as shown in figure 1*a*. The event was held during the afternoon of Saturday from 16 to 24 h and the morning of Sunday from 11 to 15 h. Several activities took place within each of the stands, offered by researchers and science communicators. Their format was diverse, including games, experiments, astronomic observation, debates, microtalks, workshops and even performances. The participants had very different interests, origins, backgrounds and ages and the event organizers estimated that 10 000 people visited the fair. The BP information stand was located at one of the entrances of the Parc de la Ciutadella as shown in figure 1 (spot number 1). This entrance was the most crowded access to the park. Visitors were encouraged to participate in the experiment by wandering around the fair while being tracked by an open source mobile app capable of running on a wide class of mobile phones. The raw data gathered are freely accessible from the project webpage at www.bee-path.net.

### 4.2. Stop-and-run walks and rectangular grid criteria algorithms: flights and stops

GPS locations are classified into two mutually exclusive groups: stopped and moving points. We used a two-step procedure inspired by Rhee *et al.* [30]. The algorithm firstly assigns by default a *stopped* flag to a given point. If the next point in the time sequence is further away than a certain threshold distance *R*_stop_, such a point is then flagged as *moving*, otherwise, the point remains as *stopped*. Starting and ending point locations of individual tracks are always by default *stopped* points. We chose an *R*_stop_=8 m which is larger than the estimated GPS errors (2–6 m). Electronic supplementary material justifies this choice. The second step of the procedure solely considers the locations flagged as *moving* by grouping them using the rectangular grid criteria [30]: a flight for individual *u* at time *t* is defined as the minimal sequence of *N* consecutive *moving* locations {**x**_1_,**x**_2_⋯**x**_*N*_} that fit inside the box defined by the segment **x**_*N*_−**x**_1_ and width corresponding to a parameter *R*_flight_. Figure 1 summarizes the two-step algorithm. Finally, despite the fact that the participants in the experiment had different demographic characteristics, they do not present highly distinctive population deviations in their movements (see the electronic supplementary material) and we have thus aggregated their tracks in our study, considering them as statistically equivalent.

### 4.3. Dynamic Brownian bridge movement model: the population-level utilization density

We applied a dBBMM [31,33] to each set of real, RW, PRW, CRW, CPRW and destination selection walk (DSW, see the electronic supplementary material) model tracks (excluding those with less than 11 locations), using the results to generate a population-level utilization density surface on a raster of 8 m cells placed over the study area. The dBBMM assigns probabilities based on the assumption of diffusion between observed locations. The model accounts for elapsed time as well as location error, and the dynamic version, proposed by Kranstauber *et al.* [33], allows the diffusion coefficient to vary depending on the variance of the trajectory observed through a sliding window. We set the location error for all points at 4.071 m, the mean of the GPS accuracy in the real data, and we used a sliding window of 11 points for the trajectory variance. We implemented the dBBMM calculations using the Move package by M. Smolla and B. Kranstauber (2015) for R (http://www.R-project.org). We also calculated the home range (95% and 99% isopleths) of each probability landscape, using the geospatial modelling environment v. 0.7.2.1. Complementary GIS processes were done using ArcGis v. 10.1 (ESRI, ArcGIS 10 online help http://help.arcgis.com/en/arcgisdesktop/10.0/help/). Maps were done using the ortophotomap 1.5000 owned by the Catalan Cartographic Institute (ICC) and available at www.icc.cat.

### 4.4. Bee-Path framework model simulations

Simulations take place inside an area of 330×145 m displayed in figure 1. Attraction wells are placed in the same configuration as the stands in the fair, creating a potential-well landscape. Each of the four proposed scenarios simulate 100 000 s (27.78 h) of real experiment. Simulation timestep Δ*t* is set at 0.1 s but individuals’ locations are sampled every 15 s and fed into the aggregation algorithm for their analysis, mimicking the data-gathering process of the real experiment. Simulated walkers are created at the same spot where participants downloaded the GPS application in the fair (spot 1 in figure 1). The number of walkers and, consequently, the number of points from where distributions are built are different, because the total simulation time is fixed and a new walker is created when the previous is removed. The numbers for RW scenario are 808 walkers with 66 781 simulated points, for PRW scenario 371 walkers and 66 726 points, for CRW 17 509 walkers and 82 581 points, and for CPRW 398 walkers and 66 712 simulated points. The value for the lifetime of each simulated walker is taken randomly from the real lifetime values of the participants of the experiment. When an individual has reached his lifetime, has visited all the wells or has crossed the borders, it is removed and another one is created following the same procedure.

## Supplementary Material

Supplementary Information: *Active* and *reactive* behaviour in human mobility: the influence of attraction points on pedestrians

## Data accessibility

The associated data of this study are openly accessible on Dryad online data repository at http://dx.doi.org/10.5061/dryad.7sj4h.
