# Supplementary material for: Active and reactive behaviour in human mobility: the influence of attraction points on pedestrians
Source: R Soc Open Sci. 2016 Jul 13;3(7):160177. doi: 10.1098/rsos.160177 (PMC4968466; doi:10.1098/rsos.160177)
Supplement: Supplementary Information: Active and reactive behaviour in human mobility: the influence of attraction points on pedestrians [file rsos160177supp1.pdf]

Supplementary Information: *Active* and *reactive* behaviour in human mobility: the influence of attraction points on pedestrians

Mario Gutiérrez-Roig,[1,*] Oleguer Sagarra,[2,†] Aitana Oltra,[2] John R.B. Palmer,[3] Frederic Bartumeus,[3] Albert Díaz-Guilera,[3] and Josep Perelló[3]

[1]*Departament de Física Fonamental, Universitat de Barcelona. Martí i Franqués 1, E-08028 Barcelona, Spain*
[2]*Centre d'Estudis Avançats de Blanes (CEAB), CSIC, Accés a la Cala Sant Francesc, 17300 Blanes (Girona), Spain*
[3]*Centre de Recerca Ecològica i Aplicacions Forestals (CREAF), Campus de Bellaterra (UAB) Edifici C, 08193 Cerdanyola del Vallès, Spain*

[*]mariogutierrezroig@ub.edu
[†]osagarra@ub.edu

CONTENTS

I. Database: Filtering procedure and population homogeneity 2

II. Movement processing algorithm 3
A. Biases generated under a random walk: Analytical treatment 4

III. Parameter selection 6
A. Potential-driven Random Walk Parameter Fit 6
B. Correlated Potential-driven Random Walk Parameter Fit 8
C. Random Walk and Correlated Random Walk Parameter fit 8

IV. *Active* and *Reactive* components 9
A. Quantifying *active* and *reactive* contributions 9
B. Simulation Results 10

V. The utilisation density and home range isopleths: More results 10

VI. Additional motion related metrics: Velocities and complete stop length distribution 11

VII. Extended analysis of long flights 11

References 15

I. DATABASE: FILTERING PROCEDURE AND POPULATION HOMOGENEITY

The raw data collected fills two tables: `userInfo` and `paths`.

1. Table userInfo: Table with one entry per usage of the app, with subject id (int), gender (char), age-group (int), move-alone flag (char), mobile-platform (char), timestamp-start (char timedate format) and timestamp-end (char timedate format). The fields are self-explanatory by their names.

2. Table paths: Contains the GPS updates labelled by individual id (int), id-user (int), latitude (decimal), longitude (decimal), timestamps (datetime char) and accuracy (float, as given by the phone).

The raw data gathered is available from the project webpage `www.bee-path.net` in two databases: `beepath_raw.sql` and `beepath_filtered.sql` which correspond to the raw and filtered datasets respectively. In the following we describe the cleaning procedure.

The initial dataset consists of 101 participants' entries and 10 312 location updates. We remove locations with accuracy values of over 6 m, as well as those with values of zero (the latter indicating failure of the GPS receiver to detect any satellites) and we remove data from subjects with less than 4 updates ($\sim$ 1 minute of observations). The dataset is further cleaned by applying a velocity thresholding procedure: If any instantaneous velocity is over 50 km/h, the end point of such trip is automatically excluded and the velocity recomputed with the next point.

From the final group of participants and updates obtained, we have performed a check of participants' homogeneity in terms of the movement statistics computed in Table S1. The results indicate that one can safely aggregate the accumulated statistics for the analysis due to the small population dispersion around the mean.

The recruitment of the participants was not homogeneous in time, as one needs to consider that the experiment was carried out during two consecutive days with different schedules. Figure S1 shows the frequency of recorded points in the server $N(h)$ as a function of the hour of the day h. We observe peaks of activity during Saturday afternoon and specially Sunday midday, which coincide with one would expect to be the busiest timeframe of the Fair, for which no additional temporal quantifiable information of attendance is available.

Attribute f	$\bar{f} \pm \sigma_f$	Median f_M
Update freq. ν (s)	28 ± 22	18
Average accuracy (m)	4 ± 1	4
Number of updates per subject N_{points}	185 ± 144	124
Total time per subject T (minutes)	61 ± 39	56
Number of Flights	13 ± 8	11
Number of Stops	11 ± 6	9
Flight velocity v(m/s)	0.61 ± 0.15	0.60
Flight length Δr_f (m)	37 ± 11	34
Stop duration Δt_s (s)	252 ± 192	246

TABLE S1. General population statistics on dataset.

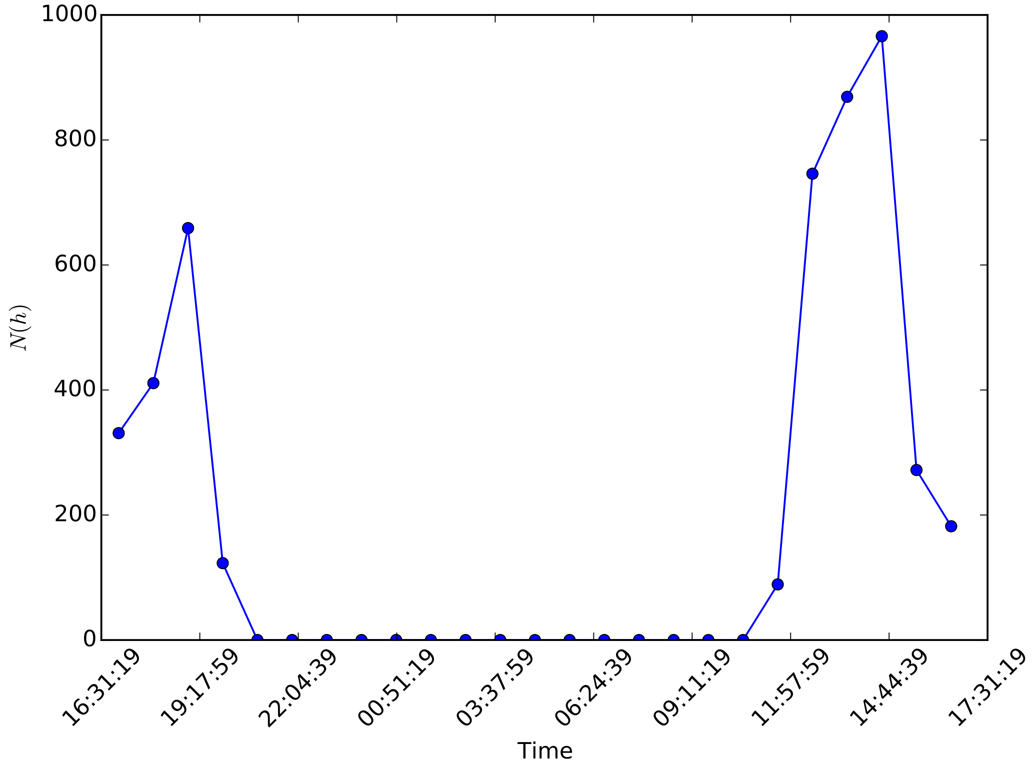

FIG. S1. Number of recorded entries per hour in the dataset (unfiltered points).

II. MOVEMENT PROCESSING ALGORITHM

Our movement processing algorithm requires two parameters (R_{stop} and R_{flight}) for splitting each participant's path into a series of flights and stops. As explained in the main text, the parameter R_{stop} sets the threshold distance that distinguishes whether a subject is moving or not. In order to fit this parameter, it is interesting to see how the fraction of GPS points that are considered "not in movement" grows as R_{stop} increases (Fig. S2, left). For the real data, we observe an elbow-shaped curve with a appreciable change of slope around the position $R_{stop} \simeq 8$ m. This is confirmed by the second derivative test, which locates the inflexion point at this position. This value for R_{stop} is coherent with the GPS accuracy: All points after filtering have an associated

accuracy value below 8 m (accuracy average is 4 m).

To test whether the *stop-and-run* algorithm induces any kind of distinctive bias, we also show the results of applying the algorithm with increasing threshold R_{stop} to the various dynamics presented in the main text. Additionally, to check for border effects, we compare to the analytical calculation for the case of totally random movement (see Fig. S2, right; see section II A) finding almost perfect coincidence, except for finite time window simulation effects. We can observe the distinct features associated to the different dynamics and real data, which shows that no external biases are generated by the model. The only bias one needs to take into account is the one related with the finite lower bound on the time between updates, which is given by the update frequency ν of each subject (in principle 15 s but may slightly vary across devices). Hence, the duration of stops will be discretized in blocks of ν seconds. Figure S2 (right) shows the different growth of the average stop duration for the empirical data and the different models. In this case, there is no saturation level and each case shows different growth rates.

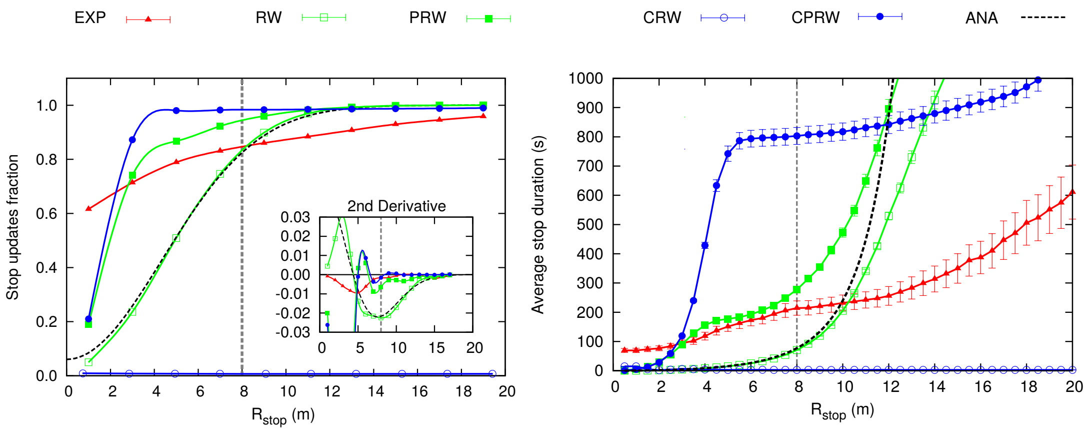

FIG. S2. (Left) Fraction of stops over total number of updates for the experiment (EXP) and the different proposed dynamics: Random Walk (RW), Potential-driven Random Walk (PRW), Correlated Random Walk (CRW), Correlated Potential-driven Random Walk (CPRW). Dashed grey line at $R_{stop} = 8$ m indicates where clear change of slope-dependence is observed for the real case. The inset shows the numerical second derivative of the previous function. (Right) Average stop duration as a function of the R_{stop} parameter for all types of motion. Dashed black line accounts for the analytical curve (ANA) of a Random Walk determined in Eq.(8) (see below).

Concerning the second step of the processing algorithm, we have chosen the value of R_{flight} in a similar manner. We have computed the average flight length dependence on R_{flight} and we have found that 8 m delimit a boundary beyond which flight length average barely changes as shown in Fig. S3. Concerning the biases induced by the flight processing algorithm, it is worth noting that due to the stopping algorithm applied, the minimal flight length is R_{stop}.

A. Biases generated under a random walk: Analytical treatment

To check for finite size and border effects in the simulations, we present here the analytical treatment for the Stops statistics under a totally Random Walk, which is used in Figs. S2 and S3.

Consider the following situation: A Brownian particle moves in an isotropic landscape. The associated Probability Density Function (PDF) corresponds to a bivariate Gaussian and reads

$$\hat{p}(x, y|t)dxdy = \frac{1}{2R_0^2\pi} \exp\left[-\frac{(x^2+y^2)}{2R_0^2}\right] dxdy. \tag{1}$$

If we represent the same distribution in polar coordinates, the marginal density for the radial

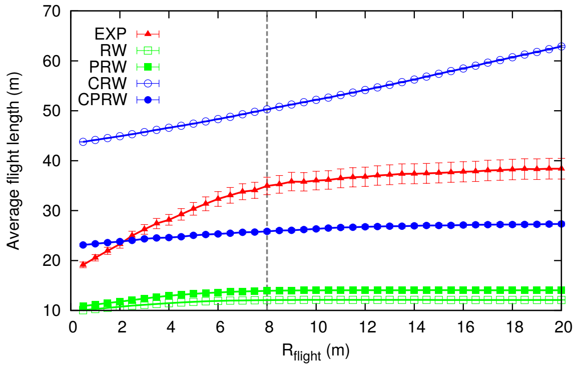

FIG. S3. Average flight length as a function of R_{flight} parameter for the experiment (EXP) and the different proposed dynamics: Random Walk (RW), Potential-driven Random Walk (PRW), Correlated Random Walk (CRW), Correlated Potential-driven Random Walk (CPRW). Dashed grey line at $R_{flight} = 8$ m indicates a shoulder in experimental data, which indicates a clear change of behaviour.

component reads

$$\hat{p}(r|t)dr = \frac{r}{R_0^2} \exp\left[-\frac{r^2}{2R_0^2}\right] dr. \tag{2}$$

In both cases, we have identified $R_0^2(t) = t/(\gamma\beta)$. The associated Cumulative Distribution Function (CDF) of the prior expression for the radial coordinates representation is

$$P(r < R_{stop}) = 1 - \int_0^{R_{stop}} \hat{p}(r|t, D)dr = 1 - \exp\left[-\frac{R_{stop}^2}{2R_0^2}\right] \equiv 1 - p(R_{stop}, R_0(t)), \tag{3}$$

which represents the probability of a particle being within a radius R_{stop} of the origin at time t.

Now consider a simulation for a long time $T = N\nu$ where the position of a Brownian particle starting at the origin is sampled every ν seconds. If ν is sufficiently separated from the integration (simulation) time τ, we can assume that the sampling will yield the same expression for the probability as in Eq. (2). In this situation, we apply our algorithm for detecting *stopped* or *moving* situations:

- We consider a point i and its successive point $i + 1$, at times t_0 and $t_0 + \tau$ respectively.
- If the two points are closer or at a distance R_{stop}, we consider that the particle is *stopped* at position $i + 1$.
- Else, we consider the particle is *moving*.

It is straightforward to see that the probability of two successive points fulfilling the conditions to be stopped is $P(r < R_{stop})$ from Eq.(3). Given that the process is Markovian (without inertia nor memory) at the time scale considered, the probabilities of successive positions being within distance R_{stop} are not correlated and equal to $1 - p$. We hence have that the probability of obtaining n_s *stopped* points (or locations) over N updates corresponds to a binomial distribution of $p = p(R_{stop}, R_0(\nu))$. We need to consider though that by default the starting point of the simulation is set to *stopped*, hence the complete expression reads,

$$P(n_s, N) = \binom{N-1}{n_s} (1-p)^{n_s} p^{N-1-n_s} \tag{4}$$

whose mean and variance are respectively

$$\langle n_s \rangle = (N-1)(1-p) + 1, \quad \text{and} \quad \sigma_{n_s}^2 = (N-1)p(1-p). \tag{5}$$

Additionally, we see that the probability of having a stop of duration $\Delta t = n\nu$ (that is: n consecutive stops) corresponds to

$$P(n) = p(1-p)^n, \tag{6}$$

whose mean and variance are now respectively

$$\langle n \rangle = \frac{1-p}{p}, \quad \text{and} \quad \sigma_n^2 = \frac{1-p}{p^2}. \tag{7}$$

Merging the expression for the mean and Eq.(3), we obtain the average stop time as a function of the parameter R_{stop} of our model. That is:

$$\langle \Delta t \rangle = \nu \langle n \rangle = \nu \left[\exp\left(\frac{R^2}{R_0^2(\nu)} \right) - 1 \right], \tag{8}$$

where we have considered that even if the particle always moves, by default it starts the simulation as *stopped*, hence $\lim_{R\to 0} \langle \Delta t \rangle = \nu$. As shown in Fig. S4, the agreement is very good.

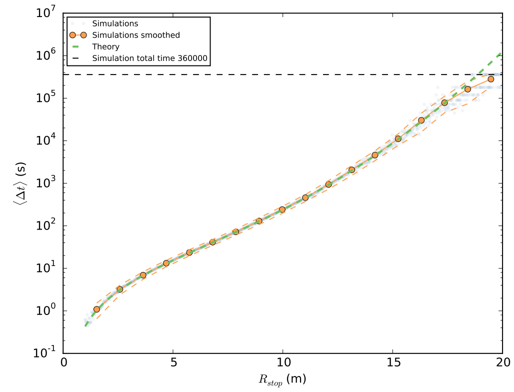

FIG. S4. Average stop length as a function of R_{flight} parameter for the Random Walk (RW) case. We see agreement between theory and simulations which is progressively lost as we approach the simulation limit time T. This is because we consider the limit $T \to \infty$ albeit obviously the maximum *stopping* time is bounded by $\Delta t \leq T$ and this fact finally induces some finite size effects in the simulation.

III. PARAMETER SELECTION

In this section we describe the method used to set all parameters for the cases presented in the main text, whose values are summarized in Table S2. For all the models, we have chosen to set the value $\gamma = 1$ as it just represents a definition of the time-scale for the process and assumes no difference among individuals. Additionally, due to the physical properties of the considered attraction poles, we have set $\sigma = 4$ m as it represents the average size of the real fair stands. This measure is also coherent with having set R_{stop} to 8 m because it makes it impossible to find a flight inside an attractive potential well, even if the subject moves from one border to the other.

A. Potential-driven Random Walk Parameter Fit

In the Potential-driven Random Walk (PRW) dynamics, there are two parameters to be fitted. V_0 is a shape property of the potential of the attraction points and the other one β accounts

Parameter	Definition	PRW	CPRW	RW	CRW
R_{stop}	Stop threshold for two consecutive updates	8m	8m	8m	8m
R_{flight}	Width of the box in rectangular model	8m	8m	8m	8m
σ	Influence distance of stands (width well)	4m	4m	4m	4m
V_0	Attractiveness of the stands (well depth)	41J	44J	41J	44J
γ	Related to resistance to forces by pedestrians	1 kg/s	1 kg/s	1 kg/s	1 kg/s
β	Temperature and diffusivity of pedestrians	1.7 m^2/s	2.6 m^2/s	1.7 m^2/s	2.6 m^2/s
κ	Degree of motion persistence (in time units)	0	7s	0	7s

TABLE S2. Summary of the parameters of the different considered dynamics: Random Walk (RW), Potential-driven Random Walk (PRW), Correlated Random Walk (CRW), Correlated Potential-driven Random Walk (CPRW). We add the corresponding dimension to each measure going thus deeper in considering human motion within framework provided by the Langevin Dynamics of particles *feeling* the attraction of a potential well.

for the dynamics of the motion. The parameters V_0 and β are set by minimizing the difference between the stop duration CDF generated by the simulation and the one observed in the real data (see Fig. S5). However, the real mobility contains two characteristic time scales, from which the interesting and distinctive part occurs for larger times. Due to the random fluctuations introduced in the model, the short stop duration statistics do not present interesting nor important differences across models, which is the main reason why we focus on long times for the parameter fit of each model. Therefore, we take into account the statistics for long stops, defined as those taking longer than 5 minutes. The k-sample Anderson-Darling (A-D) test is performed for each pair of V_0 and β in order to quantify the similarity between the distribution obtained by the simulation against the real distribution. Parameter's range is limited by values where the simulation makes sense, i.e., particles visit several attractive wells (stands) instead of getting stuck in the first one or not falling in any of them due to an excess of thermal energy.

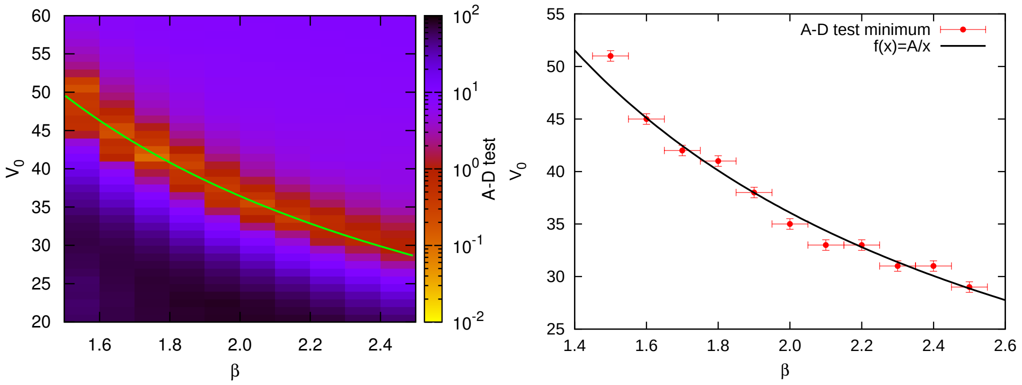

FIG. S5. Parameters fit procedure for the Potential-driven Random Walk (PRW) dynamics. (left) Heatmap of parameters fit where Anderson-Darling test value is the dependent variable. Solid green line shows the curve where A-D test value is smaller. (right) V_0 values that minimise A-D test for each parameter β. Fit of a function $f(x) = A/x$ reveals the inverse proportional relation between those variables with a constant $A = 72.16 \pm 0.56$.

Figure S5 shows how multiples pairs of values V_0 and β can successfully reproduce the exponential decaying ratio that appears in the real data. If we take the best V_0 (the one that minimises the A-D statistic) for each β we observe an inversely proportional relation between them when the decaying ratio is maintained (Fig. S5 right). This fact is qualitatively similar to the classical Kramers escape rate problem [1] but applied to a bidimensional space with multiple wells, suggesting that the probability of *escaping* an attraction pole after a certain time Δt can be approximated by an

exponential decay whose rate depends on the ratio between well depth and thermal excitation:

$$p(\Delta t) \sim \exp(-KV_0\beta\Delta t). \tag{9}$$

As expected, an increase of well attractiveness must be compensated with an increase of diffusive term (decrease of β). We have thus selected for the PRW dynamics the parameter pair which corresponds to the minimum value of the Anderson-Darling statistic.

B. Correlated Potential-driven Random Walk Parameter Fit

The procedure for the Correlated Potential-driven Random Walk (CPRW) dynamics is analogous to the previous case but with an extra parameter κ accounting for the intrinsic persistence of the motion as described in the main text.

Figure S6 describes the process in two different ways. Figure S6 shows a heatmap of the A-D statistic as a function of variables V_0 and β for a collection of κ feasible values. The case is qualitatively similar to the previous model and the inverse proportionality rule between V_0 and β is maintained. In order to select one value for κ, we plot the minimum cell value on each heatmap against κ. A U-shape curve is clearly observed clearly in Fig. S7. We then choose the minimum value, $\kappa = 7$ and its corresponding pair of parameters, $V_0 = 44$ and $\beta = 2.6$.

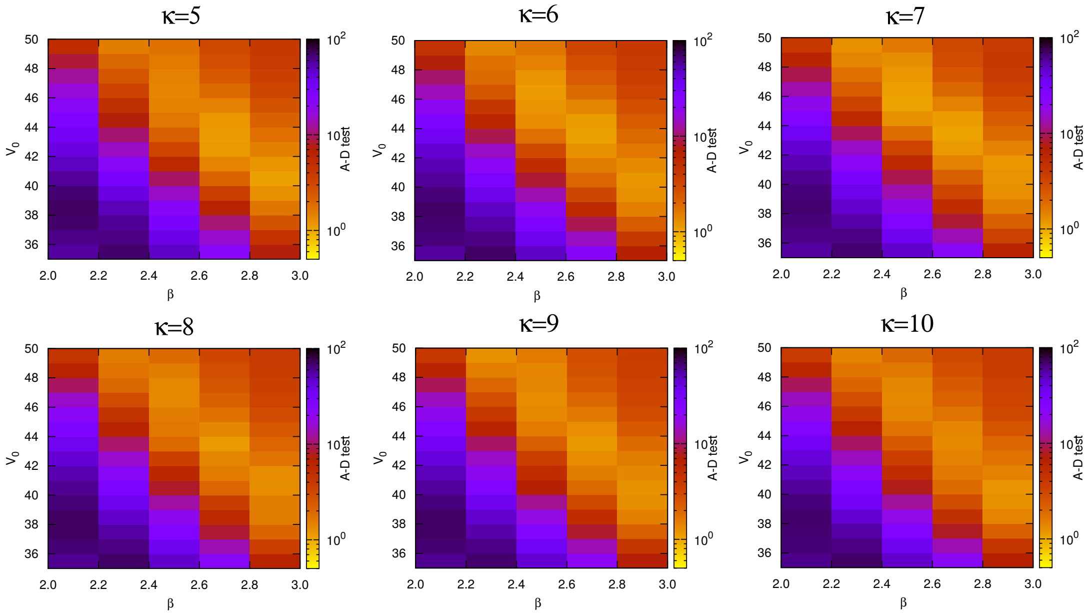

FIG. S6. Heatmap for parameters fit for the CPRW dynamics. The A-D test statistic value is provided as a function of V_0 and β. Each figure shows the result for a different κ. Parameters V_0 and β also shows inverse proportionally with constants $A_5 = 113.84 \pm 1.23$, $A_6 = 113.36 \pm 1.23$, $A_7 = 114.31 \pm 1.42$, $A_8 = 112.86 \pm 0.93$, $A_9 = 114.41 \pm 1.52$ and $A_{10} = 113.44 \pm 1.30$ respectively.

C. Random Walk and Correlated Random Walk Parameter fit

For the Random Walk (RW) and Correlated Random Walk (CRW) cases, we have used the values obtained for the PRW and the CPRW since RW and CRW are considered as null models which allow to assess and quantify the influence of the introduction of attraction poles in the walks.

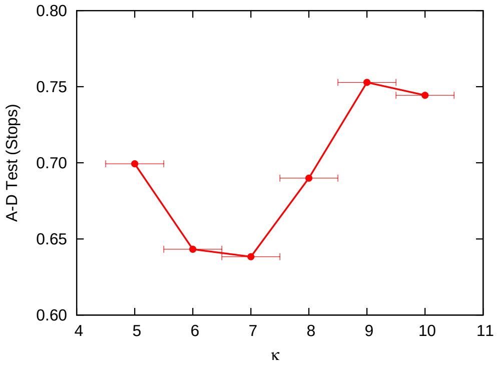

FIG. S7. Best κ fit for CPRW dynamics. We present the minimum values of A-D test statistic determined by a pair of V_0 and β values for each κ heatmap.

IV. *ACTIVE* AND *REACTIVE* COMPONENTS

The relative weight of the intrinsic motion patterns (*active* component) and landscape influence (*reactive* component) has been a frequent research issue inside motion theory and its modelling [2]. To compute their relative importance from real data is intrinsically impossible because the direct observations contain both phenomena interlinked. However, our model framework allows to quantify it by using dynamic related magnitudes.

A. Quantifying *active* and *reactive* contributions

To quantify the relative influence between *active* and *reactive* components, we compute the power P (energy or work W consumed per unit time) dissipated by pedestrians under the various dynamics proposed. To do so, we consider that individuals follow a trajectory represented by Ξ, starting at time $t = 0$ and ending at time $t = T$. This trajectory is discrete in intervals of Δt and driven by the equation (see main text),

$$\mathbf{v}(t_i) = \frac{\Delta \mathbf{r}(t_i)}{\Delta t} = \gamma^{-1}\mathbf{F}_R(\mathbf{r}(t_i)) + \gamma^{-1}\mathbf{F}_A(\mathbf{r}(t_i)), \tag{10}$$

where we identified $\mathbf{F}_R = \nabla V(\mathbf{r}(t_i), \Xi(t_i))$ and $\mathbf{F}_A = \sqrt{\frac{\gamma}{\Delta t \beta}}\rho(\mathbf{r}(t_i))\hat{\mathbf{u}}$. Based on the resulting trajectory we can compute the work W_{tot} being the energy consumed in a given trajectory Ξ. The work is defined as the sum of the contributions in each timestep

$$W_{tot} = \sum_{i=0}^{i=T/\Delta t} \Delta W_i,$$

and in which

$$\Delta W_i = (\mathbf{F}_R + \mathbf{F}_A)\Delta \mathbf{r}_i = (\mathbf{F}_R + \mathbf{F}_A)\mathbf{v}(t_i)\Delta t.$$

The work W_{tot} therefore has again two distinct sources: the *reactive* component (W_R) and the *active* component (W_A).

To go further, we therefore need to calculate the two contributions to the work. The velocity $\mathbf{v}(t_i)$ can in turn be represented again in terms of the sum of forces $\mathbf{F}_R^i + \mathbf{F}_A^i$ as shown in Eq. (10).

The two work contributions can be finally written as

$$W_R = \Delta t\gamma^{-1}\sum_i \mathbf{F}_R^i\left(\mathbf{F}_R^i + \mathbf{F}_A^i\right) = \Delta t\gamma^{-1}\sum_i (F_R^i)^2\left(1 + \frac{F_A}{F_R}\hat{\mathbf{F}}_R\hat{\mathbf{F}}_A\right)$$
$$W_A = \Delta t\gamma^{-1}\sum_i \mathbf{F}_A^i\left(\mathbf{F}_R^i + \mathbf{F}_A^i\right) = \Delta t\gamma^{-1}\sum_i (F_A^i)^2(1 + \frac{F_R}{F_A}\hat{\mathbf{F}}_R\hat{\mathbf{F}}_A). \quad (11)$$

Finally we need the work per unit time in order to make no distinction among short and long tracks. The average power of the given trajectory is then

$$\langle P\rangle = \frac{W_{tot}}{T}, \quad (12)$$

where T is the time lapse of the given trajectory. We are interested on the relative contribution of each magnitude to the power but the trajectory time T is the same, so the important magnitudes are solely the work contributions. However, the second term on the sum for both cases can have a negative sign, hence, to compute the relative importance of each component we finally use

$$\frac{|W_A|}{|W_R| + |W_A|}. \quad (13)$$

This is exactly the value provided in the main paper averaged over all paths being simulated.

B. Simulation Results

In order to evaluate *active* and *reactive* contributions in the model we run a long simulation ($T_{max} = 70\,000$ s) where we compute both *active* and *reactive* contributions in each timestep and for each trial. In the null models RW and CRW which do not incorporate potential landscape, the motion is produced only by the *active* component. Therefore, we only compute the relative weight for PRW and CPRW cases.

We present the simulation results in Fig. S8, which show two important facts: On the one hand, the proposed magnitude is well defined in both cases (it has a peaked distribution, more so in the CPRW case) despite some outliers being detected for the CPRW case tending to larger relative weights. On the other hand, the correlation between trajectory time and average relative weight shows how, in general, this measure is very stable and does not depend on the time spent moving through the area, especially in CPRW case. However, a very small fraction of individuals who 'live' for a very small time period display a very high *active* component due to the fact that such individuals scape from the bounds without having approached to any potential well.

As a final note, it is important to note that the CPRW is able to reproduce the spatial effect of participants being trapped in wells (see next Section) despite the very low *reactive* contribution detected, which is necessary also to reproduce both the utilization density and polar orientation of flights.

V. THE UTILISATION DENSITY AND HOME RANGE ISOPLETHS: MORE RESULTS

The Methods section in the main text describes the approach used to estimate the space utilisation density and home range isopleths. Figure S9 presents the results of these calculations in more detail. The dBBMM utilisation densities are visualized for real data and for all models (RW, CRW, PRW and CPRW) using a 2.5 standard deviation colour stretch, with darker colours indicating higher use intensities. The 95% isopleths are shown in grey lines, and 99% isopleths in black lines. Attractive potential wells are indicted by stars. We there observe qualitatively different spatial occupation when the *active* component takes into consideration memory effects, as in the case of the Conditional Random Walk (see and compare second row, Fig.S9(RW) vs. S9(CRW)). We also

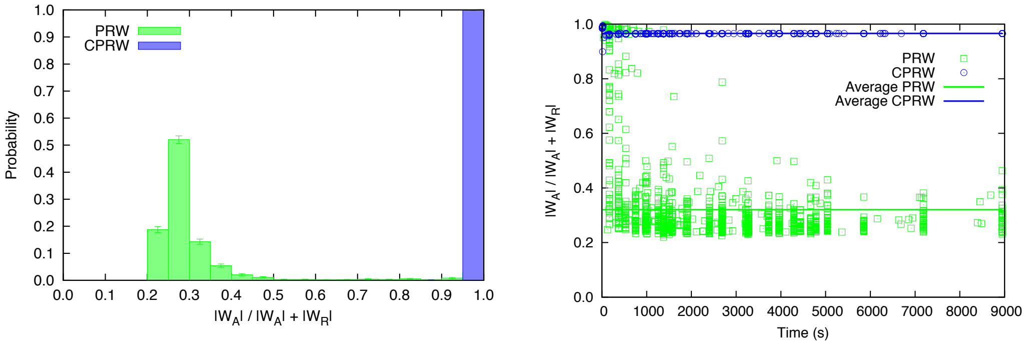

FIG. S8. (left) Probability distribution of the relative weight of *active* component for the Potential-driven Random Walk (PRW, green) and Correlated Potential driven Random Walk (CPRW, blue). (right) Relative weight of the active component as a function of life time of individuals. Solid green and blue lines mark the average for all individuals placed at 0.3206 ± 0.1542 for the PRW dynamics and 0.9659 ± 0.0051 for the CPRW dynamics.

observe in the same figure that the inclusion of the attraction potential wells (*reactive* component) in the Bee-Path framework models deeply modifies the dynamics (see Fig. S9(PRW) and Fig. S9(CPRW)).

VI. ADDITIONAL MOTION RELATED METRICS: VELOCITIES AND COMPLETE STOP LENGTH DISTRIBUTION

In addition to the characteristics studied in the main paper, we report here the flight velocity distributions for the four different dynamics considered. Figure S10 shows the distribution of flight velocities. The velocity of flights for experimental data is concentrated around a mean value which lies below standard human walking velocity ($\langle v \rangle \pm \sigma_v = 0.6 \pm 0.3$ m/s), a fact explained by the presence of attention attractors for the walkers. RW and PRW are the scenarios that better explains the empirical velocity distribution. In contrast, CRW presents a distribution centered in higher values, far above the EXP curve. The explanation for this difference is the effect of the persistence in the movement has in the rectangular grid criteria as straight movements allow to travel a same distance in less time. Consequently, the faster motion in CPRW, described by the shoulder at the right of the peak, is provoked by the same increase in velocity in the regions that are not close to any well.

In the main text, we also present the complete Stop duration distribution which shows two important timescales. The PRW is able to very well capture the two detected timescales of the model in the whole range. However, the necessary introduction of correlation in the motion leading to the CPRW breaks this feature and only reproduces the long time decay rate (slope) present in real data and associated to the stands activity.

VII. EXTENDED ANALYSIS OF LONG FLIGHTS

An important and distinct feature of the mobility detected in the experiment is the presence of unusually frequent long flights. In this section we provide an extended analysis on this motion feature. We have defined a *long flight* as a flight exceeding an Euclidean distance of 60 m, which corresponds to about 3 times the typical distance between attraction poles (19 m). There are 49 long flights in the data set performed by 24 different participants, and on average each subject performs 1.8 ± 1.4 of such flights, with the median being placed at 2 and a maximum of 6 flights per subject. The average fraction of long flights compared to the total flight performed per subject reads $\bar{f}_{LF} = 0.18 \pm 0.10$, indicating that such flights are well distributed among the population,

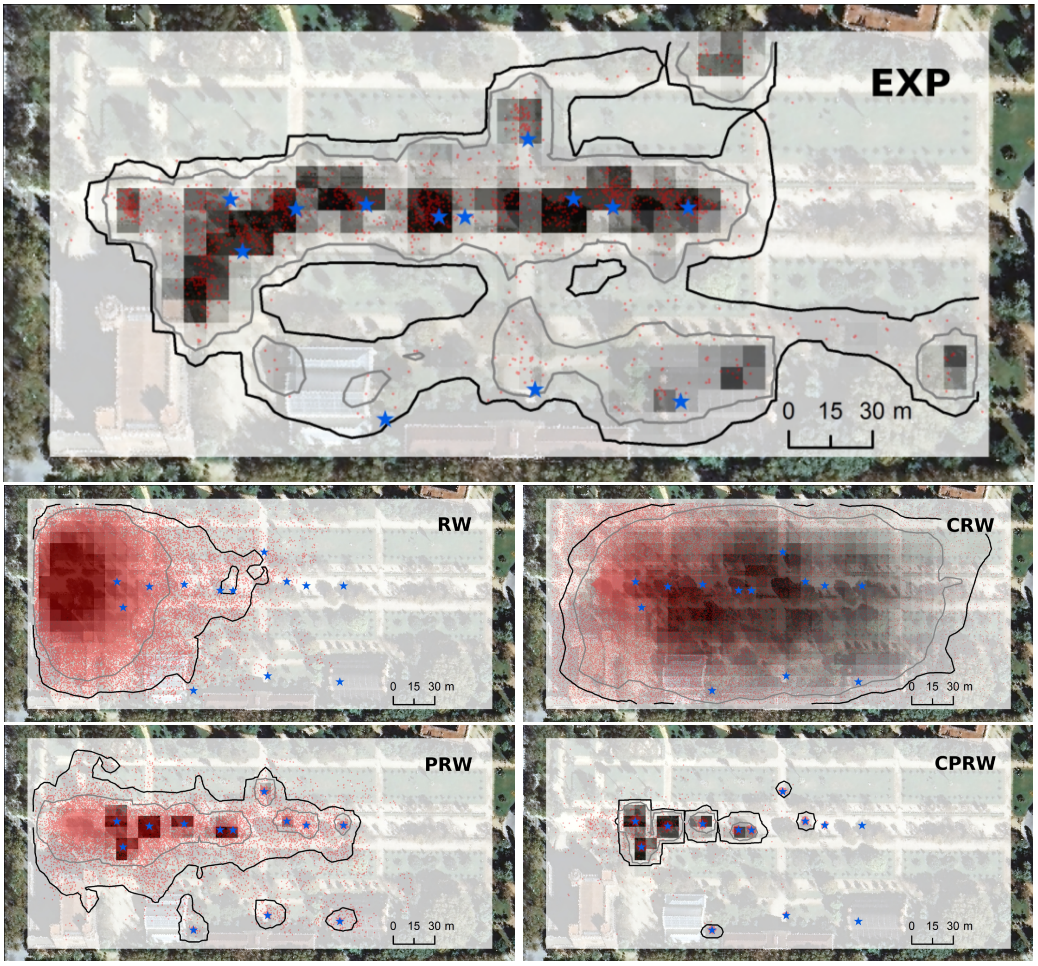

FIG. S9. Utilisation density and home range isopleths for real data and for all models based on dBBMM. First row shows empirical raw data (EXP). Second row shows two null models with no attractive wells: the Random Walk (RW) and the Conditional Random Walk (CRW). Third row shows those extensions considering the presence of attractive potential wells when intrinsic (*active*) dynamics follow a Random Walk (PRW) and a Conditional Random Walk (CPRW).

hence, they are not an artefact caused by few participants displaying uncommon behaviour. We have also studied the spatial distribution of flights, which is not isotropic but highly concentrated in the promenade zone of the fair (see Fig. S12). This spatial anisotropy may suggest a certain tendency of participants for destination selection.

The proposed framework naturally allows to test whether this feature can be solely explained by the *reactive* components. In order to reproduce this fact in our simulations, we slightly modify the model by introducing a probability to remove the influence of certain wells to a certain subject at the moment of its creation (we take and modify the CPRW and we call it Destination Selection Walk, DSW). So that, every individual does not *feel* the same amount of wells nor, consequently, the same potential landscape. The main results for the motion observables are shown in Fig. S14. The decay rate of flight length distribution is reduced with respect to the CPRW and approaches to CRW as we increase the probability of skipping wells. However, such an increase reduces the spatial coherence of the model, as the isotropy of the *perceptual* landscape is progressively recovered. The inability of the extended model to capture additional motion patterns shows how on the one hand, perceptual landscape is a necessary ingredient to model human motion, while at the same time decision-making process to select a destination is complex and cannot solely be modelled by a

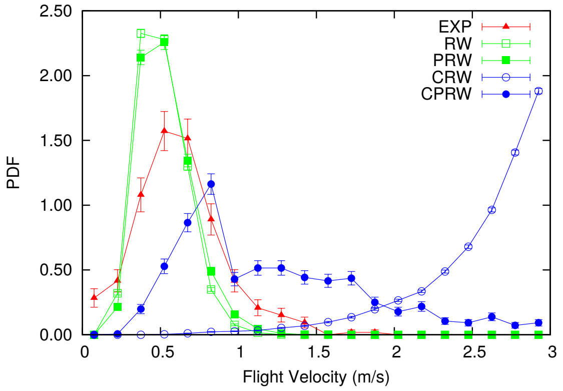

FIG. S10. Flight velocity distribution for the different proposed dynamics (RW, PRW, CRW, CPRW) in several colours and experimental data (EXP) in red.

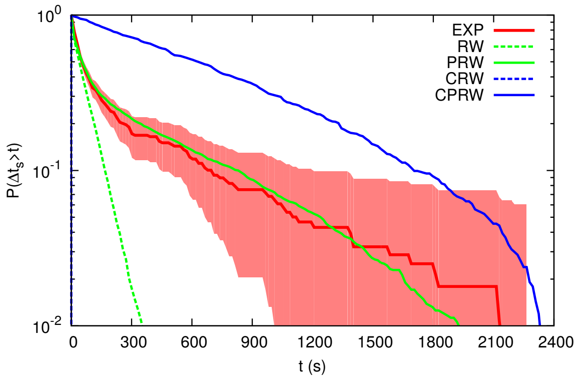

FIG. S11. Complete stop duration distribution (CDF) for the different proposed dynamics (RW, PRW, CRW, CPRW) in several colours and experimental data (EXP) in red.

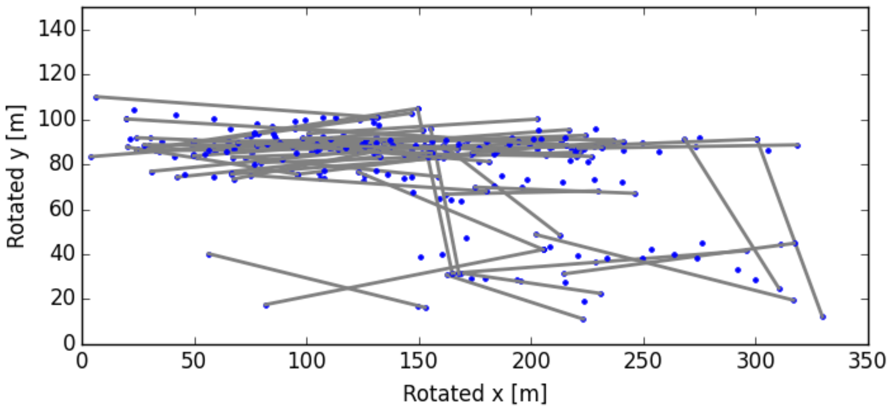

FIG. S12. Long flights spatial distribution in the fair, which are concentrated around the promenade.

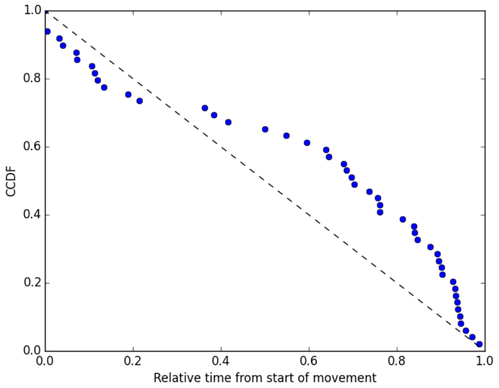

FIG. S13. Experimental long flight starting time compared to total individuals trajectory time. The dashed line corresponds to a homogeneous distribution of long flights in time.

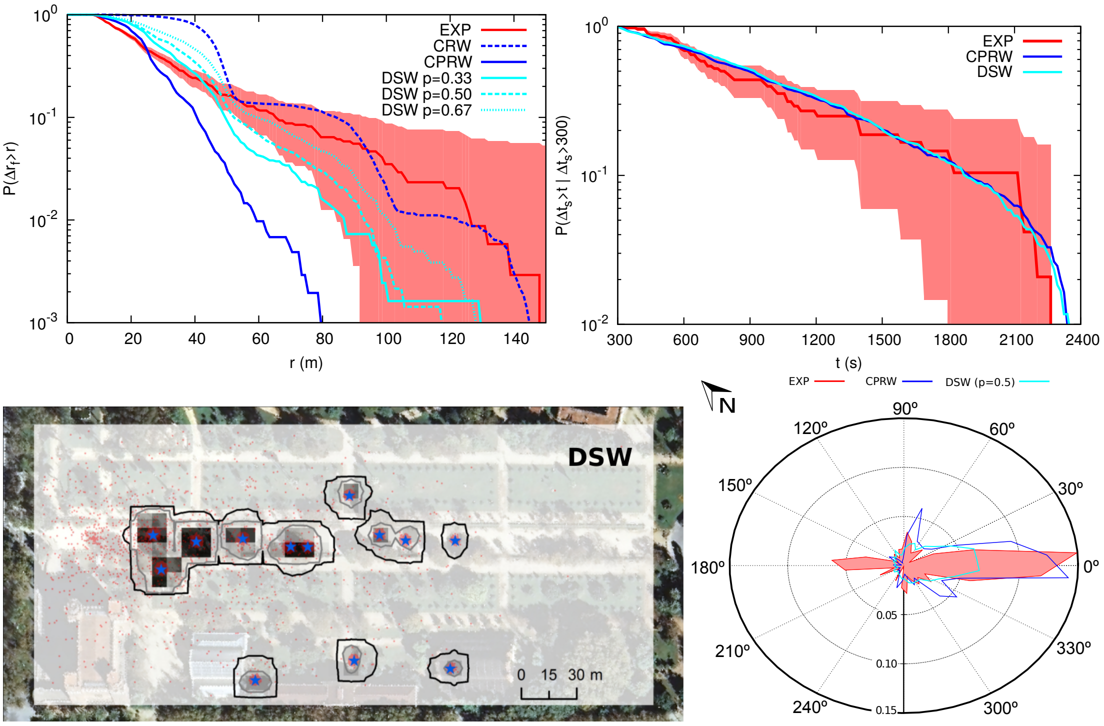

FIG. S14. Basic comparison among the Destination Selection Walk (DSW, magenta), the Correlated Potential-driven Random Walk (CPRW, blue) and the experimental results (EXP, red). (top left) Flight length distribution (CDF), (top right) stop duration distribution (CDF), (bottom left) utilisation density, and (bottom) polar plot. The DSW has a 0.5 destination selection probability.

correlation in the walkers successive steps.

The issue of destination selection is still open and our results suggest that two options are plausible to explain the presence of long flights in the experiment: Either it is caused by crowd effects (that are not accounted in the model but could be added in the form of pairwise interactions) or by some orientational bias introduced by the overall fair spatial design and stand distribution. The latter option is the most plausible due to two factors. On one hand, long flights are concentrated

in the promenade, from which participants started and ended the experiment. On the other hand, long flights tend to be concentrated towards the end of the participants' tracks (Fig. S13) which may indeed suggest that the observed motions may be associated by the fact that some participants were coming back to our stand (initial point of the experiment) to receive some feedback about the track generated and its visualization in a large screen we had for this purpose.

[1] Risken, H. *The Fokker-Planck Equation: Methods of Solution and Applications* (Springer, Berlin, 2nd Edition, 1996).

[2] Codling, E., Plank, M.J., & Benhamou, S., Random walk models in biology. *Journal of the Royal Society, Interface* **5**, 813–834 (2008).
